# Three-Dimensional Evaluation of Posterior Pole and Optic Nerve Head in Myopes with Glaucoma

**DOI:** 10.1038/s41598-017-18297-8

**Published:** 2017-12-21

**Authors:** Yong Chan Kim, Kyoung In Jung, Hae-Young Lopilly Park, Chan Kee Park

**Affiliations:** Department of Ophthalmology, Seoul St. Mary’s Hospital, College of medicine, The Catholic University of Korea, Seoul, Korea

## Abstract

The degree of myopia is represented by a global index, such as refractive error or axial length. However, the progression of myopia mainly develops in the posterior eyeball. Therefore, it is reasonable to assume that the evaluation of myopia should be confined to the posterior segment, where most of the growth and lengthening occurs. Swept source optical coherence tomography software can reconstruct the scans to the coronal view of the posterior pole, which provides additional anterior-posterior depth (z axis in the Cartesian coordinates) that is not provided with the common fundus photograph. We deduced that the parameter of deepest point of the eyeball (DPE) as a surrogate for posterior pole configuration. Between myopes with and without normal tension glaucoma (NTG) with similar axial length, myopes with NTG had deeper and more distant location of the DPE from the optic disc. The difference of the DPE position between the myopes with and without NTG may have implications for the larger optic disc tilt and torsion characteristic of myopes with NTG. Furthermore, these data suggest that myopes with NTG go through excessive posterior scleral remodeling, which may result in vulnerable optic nerve head.

## Introduction

Myopia is becoming a major public health concern among East Asian populations^1–3^. To date, research on the relationship between myopia and glaucoma has been inconclusive. There is evidence that myopia may, or may not, be a risk factor for the development and progression of glaucomatous optic nerve damage^4,5^. Investigations that reports the insignificant association between myopia and glaucoma used a global index on the severity of myopia such as refractive error or axial length^6–8^. Although refractive error and axial length are generally considered gold standards for characterizing the degree of myopia, they do not reflect precisely how the optic nerve head (ONH) is affected in the progression of myopia.

Unlike secondary high myopia due to congenital glaucoma, the axial elongation of primary myopia develops primarily in the posterior part of the eyeball^9,10^. Histologic findings at the retro-equatorial region of myopic eyes have revealed comparatively reduced density of the retinal pigment epithelium (RPE) cells compared to other regions of the eyeball^11^. In contrast, RPE density and retinal thickness in the macular region are independent of axial length, leading to the hypothesis that axial elongation chiefly occurs in the retro-equatorial region resulting in a tube-like enlargement of the globe^11,12^. Therefore, it is reasonable to assume that evaluating myopia should be confined to the posterior segment of the globe, where most of the growth and lengthening occurs.

Accordingly, our group previously investigated the posterior pole with various conventional imaging tools such as common fundus photograph or swept-source optical coherence tomography (SSOCT) or B-scans and attempted to find an association with the ONH configuration^13–15^. However, these tools were limited by providing only two-dimensional measurements of the posterior pole, and therefore could not assess the posterior pole as a three-dimensional structure. In an attempt to resolve this deficiency, we recently introduced a method to describe the three-dimensional structure of the posterior pole and used it to verify the relationship with the optic disc tilt and torsion^16^. Identifying the deepest point could be a significant factor affecting the ONH configuration because the optic disc would likely be leaned towards the direction of the DPE, and the degree of curvature would be proportional to the depth of the DPE, which showed a strong association in the non-glaucomatous emmetropic and myopic eyes^16^.

Optic disc tilt and torsion are reportedly larger in the glaucomatous myopic eyes than in non-glaucomatous myopic eyes^15,17,18^. The differences in the baseline properties of the posterior sclera may result in larger alterations in glaucomatous myopic eyes. Therefore, we compared the location of the DPE from a three-dimensional perspective in myopes with and without normal tension glaucoma (NTG). We also carried out a correlation study concerning the location of the DPE and ONH configuration in myopes with and without NTG.

## Material and Methods

This investigation was a retrospective analysis of swept source optical coherence tomography (SSOCT) scans of subjects evaluated at the glaucoma clinic of Seoul St. Mary’s Hospital between September 2016 and March 2017. This study was approved by the hospital’s institutional review board and it followed the principles of the Declaration of Helsinki and participants informed consent was obtained. Each subject received a comprehensive ophthalmologic examination at the time of the three-dimensional (3D) volumetric scan of SSOCT (DRIOCT Triton; Topcon Corporation, Tokyo, Japan). The examination included best-corrected visual acuity (BCVA), refraction, slit-lamp biomicroscopy, gonioscopy, intraocular pressure with Goldmann applanation tonometry, optic disc and red-free retinal nerve fiber layer (RNFL) photography (VX10; Kowa Optimed, Tokyo, Japan), and visual field test using the Swedish Interactive Threshold Algorithm standard 24-2 (Humphrey Visual Field Analyzer; Carl Zeiss Meditec, Inc., Dublin, CA, USA), central corneal thickness (Tomey Corporation, Nagoya, Japan), ocular biometry including anterior chamber depth (IOL Master; Carl Zeiss Meditec, Inc.), and retinal nerve fiber layer (RNFL) thickness using the aforementioned DRIOCT Triton apparatus.

A subject was excluded when eye was outside the standard configuration. The inclusion criteria were: (1) myopia when the axial length was over 24.0 mm and spherical equivalent (SE) was under -2 diopters; (2) corrected visual acuity exceeded 20/40 to minimise the effect of media opacity; and (3) open iridocorneal angle of the eye on gonioscopic examination. The exclusion criteria were: (1) history or evidence of other optic neuropathies or congenital anomalies of the optic disc; (2) signs of pathologic myopia including myopic choroidal neovascularization, lacquer crack, angioid streak; and (3) extremely myopic eyes with an axial length >30 mm. In cases in which both eyes of a subject were eligible for the study, only one eye was randomly chosen for inclusion.

For glaucoma diagnosis, patients had to fulfill the following criteria: (1) glaucomatous optic disc changes (such as localized or diffuse rim thinning, disc hemorrhage, notch in the rim, and vertical cup-to-disc ratio higher than that of the other eye by more than 0.2); (2) RNFL defects in the fundus photography or the red-free RNFL photography; and (3) glaucomatous visual field (VF) loss defined as defects corresponding to the glaucomatous structural changes. Glaucomatous VF defects were defined by glaucoma hemifield test results outside the normal limits or the presence of at least three contiguous non-edge test points within the same hemifield on the pattern deviation plot at <5%, with at least one of these points <1%, confirmed by two consecutive reliable tests (false negative <15%, false positive <15%, and fixation losses <20%); and absence of elevated IOP history >21 mm Hg. The myopic healthy group had to have no history or evidence of glaucoma, absence of elevated IOP history, or none of the above glaucoma diagnostic criteria to be included. Subjects were divided into two diagnostic groups, according to the diagnosis of glaucoma.

### Reconstruction of En face Analysis with 3D Volumetric SSOCT Scan

SSOCT using the DRIOCT Triton apparatus was done at a scan speed of 100,000 A-scans/s, center wavelength of the light beam of 1050 nm, and an effective axial resolution of 8 μm in tissue. The longer wavelength of the light beam and the less signal to noise ratio roll-off allowed a deeper scanning depth of 2.6 mm. The detailed specifications of the SSOCT have previously been described^19^. En face, or coronal scan, images are reconstructed with software provided by Topcon, using a three-dimensional volumetric scan of 12 × 9 mm, yielding a tissue imaging depth of 2.6 mm, composed of 1,000 coronal sections^20^. The en face mode provides a coronal view of the posterior segment at different depths in the anterior-posterior axis. It supplies more information than conventional cross-sectional imaging, allowing the physician to access the posterior segment beyond the retinal pigment epithelium (RPE) level in a non-invasive manner.

### Determination of the Deepest Point of the Eyeball using the En Face Images

**Figure 1:**
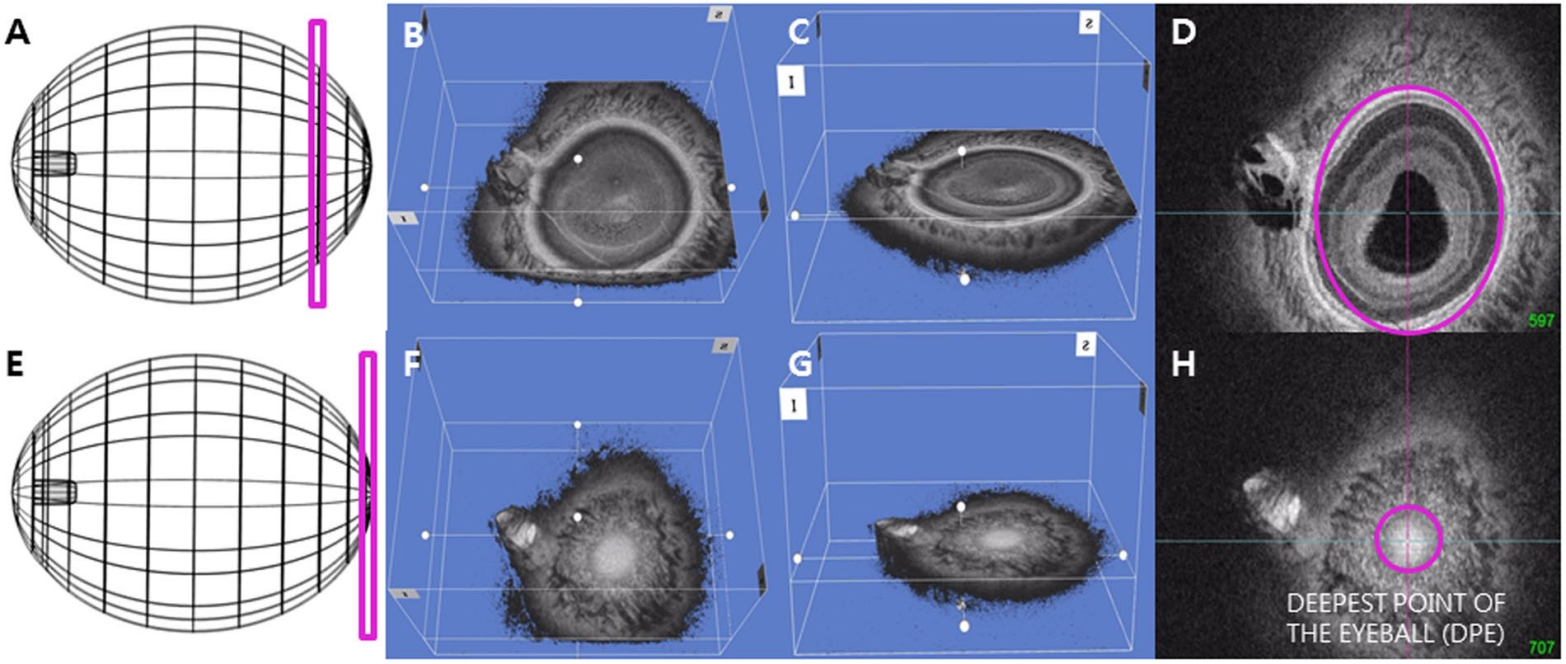
Schematic presentation of the three dimensional (3D) tomogram of the posterior pole. (**A**) Schematic figure of that sections the eyeball coronally at the purple rectangle. (**B**) En face view of the same section as A in 3D reconstruction. (**C**) Oblique view of the same section as A. (**D**) En face view of the same section in A. Purple line is overlying above the Bruch’s membrane in the same coronal section in A. (**E**) Schematic figure of that sections the eyeball coronally at the deepest point of the eyeball (DPE). (**F**) En face view of the DPE in 3D reconstruction. (**G**) Oblique view of the DPE. (**H**) En face view of the DPE. Purple line is overlying above the Bruch’s membrane at the DPE.

Details of the determination of the deepest point of the eyeball have been described previously^16^. Each en face image is the coronal view of the eyeball that is sectioned into ultrathin 2.6 μm in the anterior-posterior orientation. The interface between the different tissues can be identified by the difference of the reflectivity between the two separate tissues. Bruch’s membrane appears hyper-reflective and the choroid appears inhomogeneous and hypo-reflective in the OCT scan. Using the reflectivity difference between different structures, the interface between the different tissues can be located in the consecutive images of coronal section. The interface of the Bruch’s membrane and the choroid in the en face images appears as the hyper-reflective round plane that is surrounded by the choroid, indicating the deepest point of the eyeball (DPE) in anterior-posterior axis. This location was defined as the DPE of the posterior pole contour (Fig. 1).

### Determination of the Posterior Pole Configuration with DPE position

**Figure 2:**
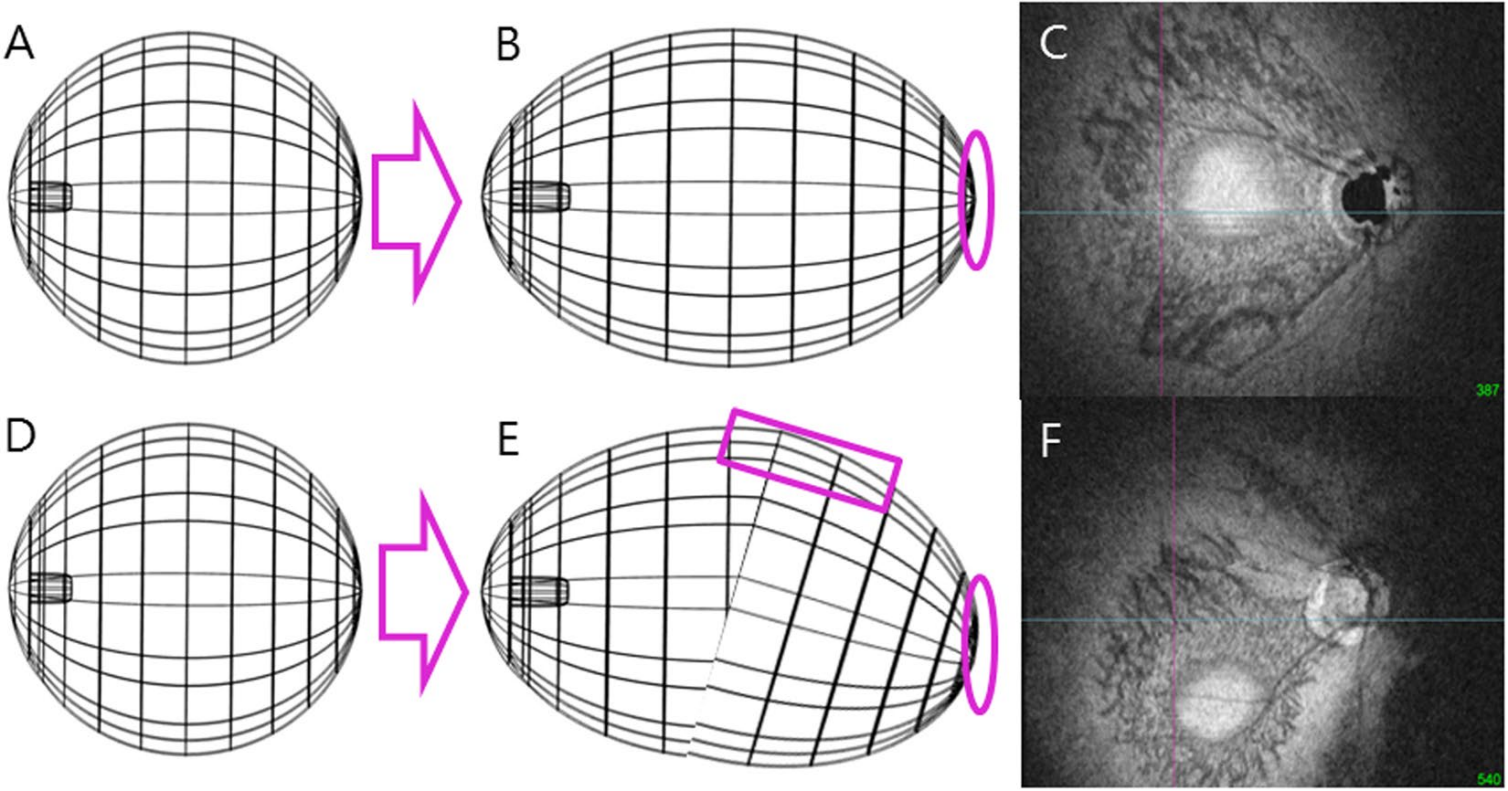
A schematic presentation of the suggested pathogenesis of the axial elongation and DPE position change. (**A**,**D**) The three dimensional eyeball appearance at baseline. (**B**,**C**) Symmetrical elongation primarily at the retro-equatorial region results in a tube-like enlargement of the globe, resulting the DPE location near the fovea. (**E**) Asymmetric growth in the retro-equatorial region of the globe results in a winding tube-like enlargement. (**F**) In this process, the DPE position is migrated towards the inferior hemisphere.

**Figure 3:**
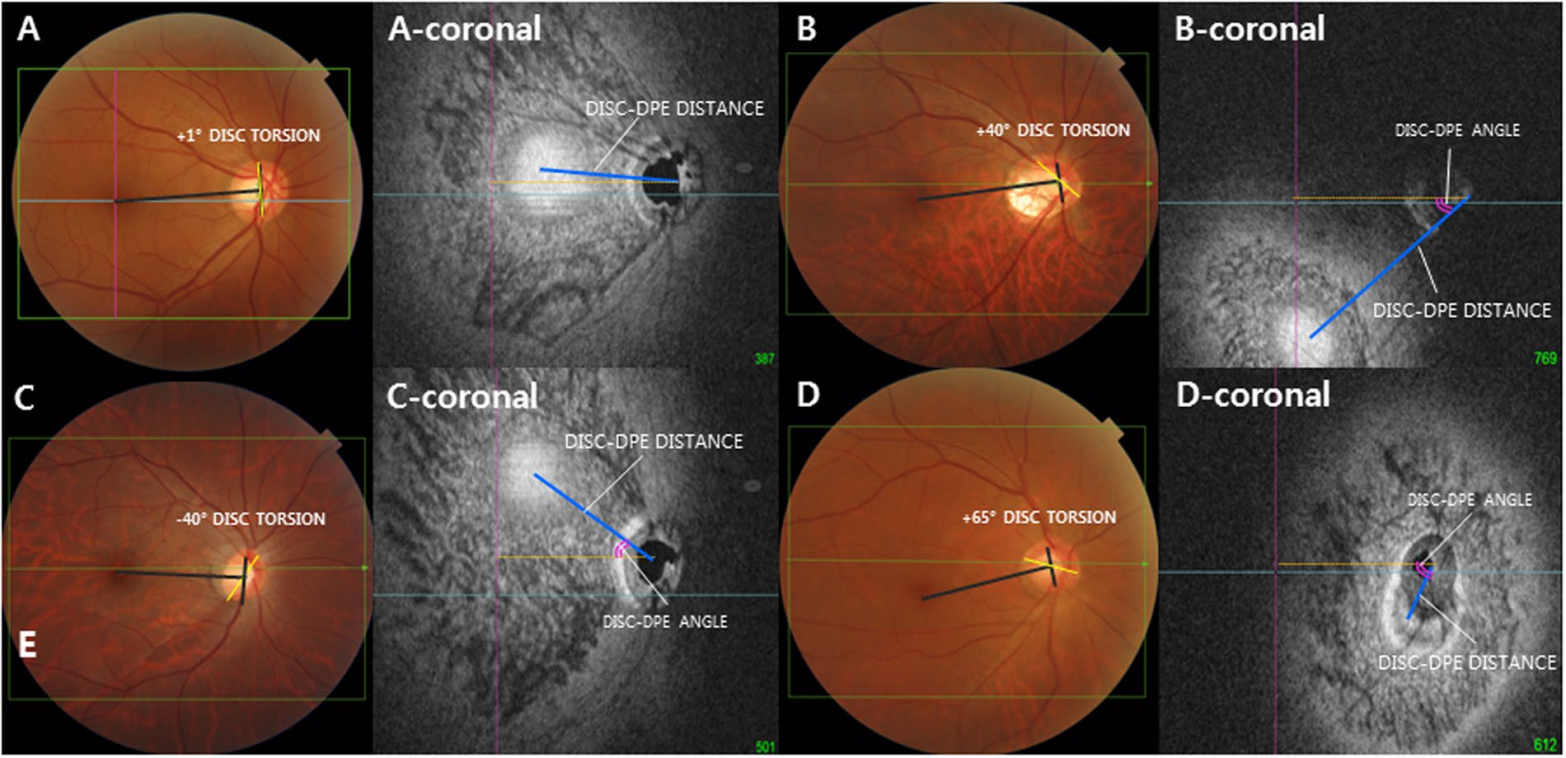
Classification of the deepest point of the eyeball (DPE) by location in the coronal plane. (**A**–**D**) Optic disc photography measuring the optic disc torsion degree. Disc torsion was identified and defined as the deviation of the long axis of the optic disc (yellow line) from the vertical meridian (dark gray line). (A-coronal) En face view of (**A**). Schematic diagram of quantifying the DPE by relative position in the coronal section. Eyes that had the center of the DPE within 3000 μm of the fovea were categorized as the near fovea group. (B-coronal) En face view of (**B**). Eyes that had the center of the DPE in the superior half of the globe, based on an imaginary line connecting the fovea and the center of the disc and excluding the fovea and disc area, were categorized as the superior hemisphere group. (C-coronal) En face view of (**C**). Eyes that had the center of the DPE in the inferior half of the globe, based on the imaginary line connecting the fovea and the center of the disc and excluding the fovea and disc area were categorized as the inferior hemisphere group. (D-coronal) En face view of (**D**). Eyes that had the center of the DPE within 3000 μm of the disc were categorized as the near disc group.

**Figure 4:**
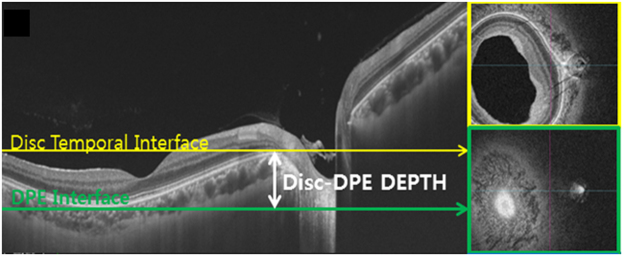
The two parameters that represents the posterior pole depth. (**A**) The Disc-DPE depth was the depth between the interface of the DPE and the interface of temporal border of the optic disc. (**B**) The Fovea-Disc depth was the depth between the interface of the fovea and the interface of temporal border of the optic disc.

Asymmetric growth in the retro-equatorial region of the eyeball would result in a winding tube-like enlargement that could alter the DPE position (Fig. 2D,E,F). The altered DPE position can be used as a surrogate to determine the posterior pole alteration, because the DPE position can reflect every axis of coordinates. The DPE location in the posterior pole was quantified by using the caliper function of the built-in software of the OCT by 2 authors in a blinded fashion (YCK and HYP). The SSOCT software provides center of the disc as a green cross based on the margin of Bruch’s membrane as a default. The Disc-DPE distance was defined as the straight line distance between the center of the optic disc to the center of the DPE measured at the same coronal plane of the DPE (Fig. 3). To quantify the angular position of the DPE relative to the optic disc, the horizontal meridian crossing the OCT-defined center of the optic disc was determined as the reference line. The Disc-DPE angle was defined as the angle between the horizontal meridian crossing the OCT-defined center of the optic disc and the straight line from the OCT-defined center of the optic disc to the DPE measured by the intrinsic caliper of the built-in software (Fig. 3). Likewise, the Disc-Fovea angle was defined as the angle between the horizontal meridian crossing the OCT-defined center of the disc and the straight line from the OCT-defined center of the optic disc the fovea^21^. The depth between the different posterior pole contour structures was calculated by the number of coronal sections between the interfaces of the different structures. After counting the depth by the separate images of the coronal section, the depth was switched into micrometers by counting each coronal section as 2.6 μm in depth. The Disc-DPE depth was the depth between the interface of the DPE and the interface of temporal border of the optic disc (Fig. 4).

To compensate for the potential errors induced by head tilt or ocular rotation, the subjects’ eyes were aligned with the eye level mark on the forehead rest support by raising or lowering the chin rest. Subjects were instructed to hold their heads in a vertical position throughout the photographic session. Using the eye to be scanned, each patient was instructed to look directly at the internal fixation target in the OCT camera. This device uses comfortable 1,050 nm scanning light and has real time eye tracking that has been known to eliminate the eye motion and minimizes artifacts by fixating on the fovea on each scan.

### Posterior Pole Classification with the DPE location

The subjects were categorized into the following four groups based on the DPE location. The near-fovea group was the eyes that had their center of DPE in a 3000 μm radius of the fovea (Fig. 3A). The inferior hemisphere group was the eyes that had their center of DPE in the inferior half of the globe based on an imaginary line connecting the fovea and the center of the disc and excluding the fovea (Fig. 3B). The superior hemisphere group was the eyes that had their center of DPE in the superior half of the globe based on an imaginary line connecting the fovea and the center of the disc and excluding the fovea (Fig. 3C). The near-disc group was the eyes that had their center of DPE in a 3000 μm radius of the disc (Fig. 3D).

### Measurement of Optic Disc Torsion

Fundus photographs were acquired using a SSOCT scan, each centered on the macula to the 30 degrees periphery. Optic disc torsion was measured from photographs by two glaucoma specialist (YCK and KIJ) with the Topcon built-in software. Disc torsion was identified and defined as a deviation of the long axis of the optic disc from the vertical meridian^22,23^. The vertical meridian was considered a vertical line 90° from a horizontal line connecting the fovea to the center of the optic disc. The angle between the vertical meridian and the longest diameter of the optic disc was considered the degree of torsion (Fig. 3). A positive torsion value indicated inferotemporal torsion (which is counterclockwise torsion in the right eye format), and a negative value indicated superonasal torsion (which is clockwise torsion in the right eye format). Details have been described previously^24^.

### Measurement of Optic Disc Tilt

Optic disc tilt was identified by three different measure, disc ovality ratio, horizontal disc tilt, and vertical disc tilt, respectively. Disc ovality ratio was defined as the ratio between the longest and shortest diameters of the optic disc (tilt ratio = LD/SD)^25^. Horizontal and vertical disc tilt angle was measured according to a method proposed by Hosseini *et al*.^26^. Briefly, a triple overly image consisted of a color fundus photograph, the en face OCT image, and the corresponding horizontal OCT scan were created. Using this layout, the clinical boundary of the disc locates the respective points on the horizontal OCT image. ONH plane was defined as the line connecting the clinical boundary of the disc on each side of the ONH. A reference plane was defined as the line connecting the inner edges of the Bruch’s membrane on each side of the ONH. The tilt angle defined as the angle between the reference and the ONH plane. Angle measurements were performed by two observers (YCK and HYP) with the software intrinsic angle tool. A positive degree of horizontal tilt indicated a temporal tilt, and a negative value indicated a nasal tilt. The degree of vertical tilt was also measured from a vertical cross-sectional image as described above. A positive degree of tilt indicated an inferior tilt, and a negative value indicated a superior tilt.

### Measurement Reproducibility

The interface between the Bruch’s membrane and the choroidal tissue was determined manually by the two blinded observers. The analysis was based on 30 independent series (15 on glaucomatous eyes and 15 on non-glaucomatous eyes) of inter-visit reproducibility conducted twice on different days by two of the authors (YCK and YJ). The intra-class correlation coefficients were determined by a two-way mixed effect model^27^. ICC scores >0.75 are considered excellent (Table 1)^28^.

**Table 1:** Reproducibility of the measurements of the deepest point of the eyeball interface, Disc-DPE distance, Disc-DPE depth, Fovea–DPE depth, and Disc-DPE angle using swept-source optical coherence tomography in myopic glaucomatous eyes and myopic non-glaucomatous eyes.

	Intraobserver ICC*		Interobserver ICC^†^
	ICC (Observer 1)	ICC (Observer 2)	
DPE interface	1.000 (0.999–1.000)	0.999 (0.999–1.000)	0.998 (0.994–0.999)
Disc-DPE distance, mm	0.975 (0.945–0.989)	0.945 (0.922–0.981)	0.926 (0.901–0.969)
Disc-DPE Depth, mm	0.998 (0.995–0.999)	0.998 (0.995–0.999)	0.998 (0.995–0.999)
Fovea-Disc Depth, mm	0.998 (0.995–0.999)	0.998 (0.995–0.999)	0.996 (0.992–0.999)
Disc–DPE angle (°)	0.965 (0.933–0.993)	0.943 (0.912–0.988)	0.955 (0.920–0.984)

DPE: deepest point of the eyeball; ICC: intraclass correlation coefficient. *ICC for single measure. ^†^ICC for average measure.

### Statistical Analysis

Statistical analyses were performed using the SPSS software version 23.0 (SPSS, Chicago, IL, USA). Independent *t*-test and one way ANOVA were used to compare the data among the groups. The χ² test was used to analyze categoric variables. The Pearson’s correlation analysis was calculated to assess the relationships of the DPE quantification and the ocular parameters. Univariate and multivariate linear regression analyses were conducted to identify the association between the posterior pole profiles and disc tilt and torsion in each group. Dependent variables were tilt and torsion, and the independent variables were Disc-DPE distance, Fovea-DPE distance, Disc-DPE depth, Fovea-DPE depth and Disc-DPE angle. The variables that retained significance at *P* < 0.2 in the univariate analysis were included in the multivariate model. The level of statistical significance was set at *P* < 0.05.

## Results

Initially, 220 healthy subjects and 191 NTG patients with myopia were enrolled. After matching the glaucoma patients and healthy subjects for age and axial length between groups, 161 NTG eyes and 178 healthy eyes remained, of which 22 were excluded because of poor scan image quality, 14 were excluded because of unreliable VF tests, and 6 were excluded because of atopic optic discs. The remaining 254 eyes (125 myopes without NTG and 129 myopes with NTG) were included in the analysis. All subjects were Korean.

The mean age of the included subjects was 45.29 ± 11.74 years (range, 14 to 82 years), the mean spherical equivalent (SE) was −4.63 ± 3.62D (range, −0.50 to −20.5D), mean axial length was 26.34 ± 1.36 mm (range, 24.02 to 30.75 mm), and mean deviation of perimetry (MD) at initial examination was −3.85 ± 4.71 dB (range, 2.35 to −26.96). In comparison between the myopes without NTG and myopes with NTG, myopic NTG eyes had greater MD (−6.18 ± 5.46 vs. −1.46 ± 1.75 dB; *P* < 0.001) and thinner RNFL (73.66 ± 14.74 vs. 94.40 ± 14.09 μm; *P* < 0.001) than the myopes without NTG. Age, SE, axial length, anterior chamber depth, and central corneal thickness were not significantly different between groups (Table 2).

**Table 2:** Baseline characteristics of the myopic non-glaucomatous eyes and the myopic glaucomatous eyes*.

	Myopes without NTG (*n* = 125)	Myopes with NTG (*n* = 129)	*P value* ^†^
Age, years	43.70 ± 11.96	44.84 ± 11.36	0.184
Laterality, right:left	55:70	77:52	**0.012** ^**§**^
Gender, male:female	51:74	62:67	0.244^§^
Spherical equivalent, diopter	−4.34 ± 3.53	−4.54 ± 3.68	0.210
Axial length, mm	26.02 ± 1.33	26.16 ± 1.32	0.212
Anterior chamber depth, mm	3.21 ± 0.64	3.06 ± 0.74	0.091
Central corneal thickness, μm	534.64 ± 45.24	524.77 ± 46.54	0.093
Visual field MD, dB	−1.46 ± 1.79	−6.18 ± 5.46	**<0.001** ^**‡**^
Average RNFL thickness, μm	94.40 ± 14.09	73.66 ± 14.74	**<0.001** ^**‡**^

*Data are presented as mean ± standard deviation unless otherwise indicated. ^†^Independent *t*-test for continuous variables. ^‡^Statistically significant values (*P* < 0.05) are shown in bold. ^§^χ² test for categorical variables.

Comparison of the posterior pole profiles and the optic disc configurations between the myopes without NTG and myopes with NTG were found to be significantly different (Table 3). The location of the DPE in the myopes with NTG group was mostly near the fovea (51.9%), followed by the near-disc, inferior, and superior positions(19.3%, 18.6%, and 5.4%, respectively), whereas in the myopes without NTG group, the majority of DPEs were found at the inferior (43.2%), followed by the near fovea, near disc, and superior positions (31.2%, 20.0%, and 5.6%, respectively); the distribution of the DPE position for the two groups was significantly different (*P* = 0.004). The Disc-DPE distance was significantly greater in glaucomatous eyes than non-glaucomatous eyes (4.01 ± 1.27 vs. 3.66 ± 1.31 mm; *P* = 0.031). The Disc-DPE depth was greater in those with glaucoma than those without (0.17 ± 0.16 vs. 1.3 ± 0.17 mm; *P* = 0.057). The fovea-Disc depth was significantly less in glaucomatous eyes than non-glaucomatous eyes (0.18 ± 0.25 vs. 0.25 ± 0.25 μm; *P* = 0.044). The disc configuration of ovality index, horizontal tilt angle, and vertical tilt angle was significantly larger in the myopes with NTG (*P* < 0.001, *P* < 0.001, and *P* = 0.035, respectively). However, the Disc-DPE angle, disc fovea angle, and disc torsion angle were not significantly different between the glaucomatous myopes and the healthy myopes (*P* = 0.896, *P* = 0.448, and *P* = 0.196, respectively) (Table 3).

**Table 3:** The comparison on the posterior pole profiles and the optic disc configuration between the myopic non-glaucomatous eyes and the myopic glaucomatous eyes*.

	Myopes without NTG (*n* = 125)	Myopes with NTG (*n* = 129)	*P value* ^†^
Location of the DPE (eyes)	39:7:54:25	67:7:31:24	
Fovea: superior: inferior: disc (%)	31.2:5.6:43.2:20.0	51.9:5.4:18.6:19.3	**0.004§**
Disc-DPE distance, mm	3.66 ± 1.31	4.01 ± 1.27	**0.031** ^**‡**^
Disc-DPE depth, mm	0.13 ± 0.17	0.17 ± 0.16	**0.044** ^**‡**^
Disc–DPE angle (°)	25.93 ± 25.81	26.41 ± 32.19	0.896
Disc–foveal angle (°)	7.36 ± 3.45	7.71 ± 3.95	0.448
Disc torsion angle (°)	15.80 ± 28.07	11.27 ± 26.14	0.196
Disc ovality index	1.24 ± 0.18	1.37 ± 0.30	**<0.001** ^**‡**^
Horizontal tilt angle (°)	12.04 ± 9.54	17.26 ± 9.33	**<0.001** ^**‡**^
Vertical tilt angle (°)	6.08 ± 10.15	8.99 ± 11.79	**0.036** ^**‡**^

DPE: deepest point of the eyeball. *Data are presented as mean ± standard deviation unless otherwise indicated. ^†^Independent *t*-test for continuous variables between the glaucomatous and healthy eye groups. ^‡^Statistically significant values (*P* < 0.05) are shown in bold. ^§^χ² test for categorical variables.

Table 4 shows the correlation analysis of the posterior pole configuration with the subject characteristics. The axial length was not significantly associated with the posterior pole configuration. Moreover, the axial length was not associated with any of the ONH configuration in myopes with NTG. The average RNFL thickness was reduced significantly with the deeper Disc-DPE depth in both myopes with NTG and without NTG. The Disc-DPE distance and the Disc-DPE depth was significantly correlated with the amount of disc ovality index in myopes with and without NTG.

**Table 4:** Comparison of posterior pole characteristics according to the different locations of the DPE in myopic non-glaucomatous patients and the myopic glaucomatous eyes*.

Variables	Disc-DPE Distance				Disc-DPE Depth				Axial length			
	Myopes without NTG		Myopes with NTG		Myopes without NTG		Myopes with NTG		Myopes without NTG		Myopes with NTG	
	*R*	*P* Value*	*R*	*P* Value*	*R*	*P* Value*	*R*	*P* Value*	*R*	*P* Value*	*R*	*P* Value*
Age, years	−0.082	0.437	−0.204	**0.036** ^**†**^	−0.094	0.371	−0.233	**0.017** ^**†**^	−0.407	**<0.001** ^**†**^	−0.191	**0.030** ^**†**^
Axial length, mm	0.111	0.288	0.141	0.152	0.054	0.593	0.151	0.125				
Visual Field MD, dB	−0.265	**0.008** ^**†**^	−0.091	0.359	−0.289	**<0.001** ^**†**^	−0.218	0.014	−0.198	0.058	−0.064	0.520
Average RNFL thickness, μm	−0.223	**0.031** ^**†**^	−0.254	**0.009** ^**†**^	−0.441	**<0.001** ^**†**^	−0.335	**0 < 0.001** ^**†**^	−0.123	0.131	−0.125	0.205
Disc-DPE distance, μm					0.763	**<0.001** ^**†**^	0.714	**<0.001** ^**†**^	0.111	0.288	0.141	0.152
Disc-DPE depth, μm	0.763	**<0.001** ^**†**^	0.714	**<0.001** ^**†**^					0.054	0.593	0.151	0.125
Disc-DPE angle (°)	−0.069	0.512	0.008	0.939	0.051	0.613	0.029	0.772	0.108	0.166	0.195	**0.027** ^**†**^
Disc-foveal angle (°)	0.047	0.654	0.112	0.256	−0.073	0.468	0.185	0.059	0.019	0.804	0.226	**0.010** ^**†**^
Disc torsion angle (°)	0.030	0.778	0.052	0.600	0.090	0.372	0.121	0.219	0.215	**0.005** ^**†**^	0.094	0.290
Disc ovality index	0.336	**<0.001** ^**†**^	0.340	**<0.001** ^**†**^	0.377	**<0.001** ^**†**^	0.472	**<0.001** ^**†**^	0.182	**0.019** ^**†**^	0.152	0.085
Horizontal tilt angle (°)	0.317	**<0.001** ^**†**^	0.151	0.087	0.182	**0.019** ^**†**^	0.132	0.134	0.134	0.120	0.066	0.459
Vertical tilt angle (°)	0.097	0.214	0.059	0.505	−0.051	0.512	0.057	0.525	0.066	0.544	0.103	0.245

Because of the significant difference in the distributions of the DPE location between the two groups, overall comparisons of the posterior pole profiles might not reflect the proper association with the optic disc configuration. Thus, a sub-analytic comparison between myopes with NTG and myopes without NTG was done separately, based on the location of the DPE in the posterior pole (Table 5). The Disc-DPE distance was significantly different only for the inferior hemisphere group in the sub-analysis (4.50 ± 0.97 vs. 3.84 ± 1.22 mm; *P* = 0.012). The sub-analytic comparison of all four groups revealed a larger mean Disc-DPE depth in myopes with NTG compared with myopes without NTG; only the comparison in the DPE near-disc group was significantly different between NTG/non-NTG (0.022 ± 0.03 vs. 0.019 ± 0.18 mm; *P* = 0.001). While the sub-analysis of all four groups showed a larger mean Disc-DPE angle in the myopes with NTG than in the myopes without NTG, only the comparison in the DPE near-disc group revealed a significant difference (59.68 ± 52.28 vs. 35.42 ± 31.93°; *P* = 0.050). The sub-analysis of the four groups revealed a larger mean Disc-fovea angle and larger mean disc torsion angle in myopes with NTG compared to myopes without NTG, with no significant differences. Every optic disc tilt index had a larger value in the myopes with NTG in all four sub-analytic groups; in the inferior hemisphere group all three indices were significantly different.

**Table 5:** Comparison of posterior pole characteristics according to the different locations of the DPE in myopic non-glaucomatous patients and the myopic glaucomatous eyes*.

	Near Fovea		*P value* ^†^	Superior Hemisphere		*P value†*	Inferior Hemisphere		*P value* ^†^	Near Disc		*P value* ^∥^
	Myopes without NTG (*n* = 125)	Myopes with NTG (*n* = 129)		Myopes without NTG (*n* = 125)	Myopes with NTG (*n* = 129)		Myopes without NTG (*n* = 125)	Myopes with NTG (*n* = 129)		Myopes without NTG (*n* = 125)	Myopes with NTG (*n* = 129)	
Number of eyes	39	67		7	7		54	31		25	24	
Inferotemporal RNFL defect, number (%)		51 (76.1%)			4 (57.1%)			22 (71.0%)			16 (66.7%)	
Age, years	42.6 ± 12.0	43.5 ± 10.2	0.183	49.5 ± 7.8	45.0 ± 10.1	0.535	43.1 ± 13.5	46.1 ± 13.7	0.113	47.4 ± 8.1	50.0 ± 9.8	**0.024** ^**‡**^
SE, diopter	−4.2 ± 3.2	−5.1 ± 3.5	0.184	−2.3 ± 1.8	−1.0 ± 1.9	0.165	−4.6 ± 4.9	−5.5 ± 3.3	0.195	−4.6 ± 4.9	−4.8 ± 4.4	0.828
Axial length, mm	26.2 ± 1.3	26.6 ± 1.1	0.128	25.3 ± 1.2	25.4 ± 1.3	1.000	26.1 ± 1.2	26.9 ± 1.4	**0.005** ^**‡**^	25.8 ± 1.3	26.8 ± 1.5	**0.009** ^**‡**^
Visual field MD, dB	−1.4 ± 1.6	−6.3 ± 4.7	**<0.001** ^**‡**^	−1.7 ± 2.2	−2.9 ± 4.1	0.318	−1.5 ± 1.9	−6.6 ± 6.3	**<0.001** ^**‡**^	−1.4 ± 1.9	−6.1 ± 6.4	**<0.001** ^**‡**^
Average RNFL thickness, μm	93.2 ± 12.9	72.9 ± 12.4	**<0.001** ^**‡**^	99.1 ± 9.8	85.1 ± 13.2	0.073	94.4 ± 13.2	74.3 ± 15.6	**<0.001** ^**‡**^	94.8 ± 18.3	71.4 ± 18.9	**<0.001** ^**‡**^
Disc-DPE distance, mm	4.54 ± 0.58	4.51 ± 0.75	0.828	3.61 ± 0.95	3.78 ± 0.77	0.620	3.84 ± 1.22	4.50 ± 0.97	**0.012** ^**‡**^	1.91 ± 0.51	2.06 ± 0.99	0.575
Disc-DPE depth, mm	0.20 ± 0.15	0.21 ± 0.14	0.882	0.064 ± 0.07	0.093 ± 0.14	0.318	0.13 ± 0.16	0.21 ± 0.21	**0.053**	0.019 ± 0.18	0.022 ± 0.03	**0.001** ^**‡**^
Disc–DPE angle (°)	12.97 ± 9.42	14.19 ± 8.86	0.504	−23.50 ± 8.17	−21.07 ± 10.52	0.620	37.31 ± 20.12	37.78 ± 15.86	0.910	35.42 ± 31.93	59.68 ± 52.28	**0.050**
Disc-foveal angle (°)	6.45 ± 3.09	7.04 ± 4.06	0.434	7.75 ± 3.40	5.65 ± 5.04	0.383	7.84 ± 3.25	9.17 ± 3.45	**0.079**	7.64 ± 4.26	8.31 ± 3.43	0.535
Disc torsion angle (°)	7.18 ± 13.58	3.61 ± 21.96	0.362	−21.43 ± 9.88	−15.00 ± 33.54	0.318	24.79 ± 26.1	27.09 ± 29.18	0.710	20.24 ± 38.74	19.67 ± 28.56	0.802
Disc ovality index	1.29 ± 0.25	1.40 ± 0.34	**0.066**	1.18 ± 0.11	1.35 ± 0.18	**0.047** ^**‡**^	1.23 ± 0.14	1.39 ± 0.28	**0.001** ^**‡**^	1.22 ± 0.15	1.23 ± 0.20	0.812
Horizontal tilt angle (°)	12.17 ± 6.97	18.34 ± 6.98	**<0.001** ^**‡**^	11.43 ± 9.74	10.85 ± 7.88	0.902	12.11 ± 8.86	16.83 ± 10.01	**0.027** ^**‡**^	11.84 ± 13.99	16.67 ± 13.48	0.117
Vertical tilt angle (°)	4.18 ± 8.73	7.34 ± 11.53	0.141	0.86 ± 2.27	3.28 ± 5.96	0.620	8.44 ± 11.38	13.16 ± 11.36	**0.015** ^**‡**^	8.44 ± 11.38	8.99 ± 11.79	0.739

SE: spherical equivalent; MD: mean deviation of perimetry; RNFL: retinal nerve fiber layer; DPE: deepest point of the eyeball. *Data are presented as mean ± standard deviation unless otherwise indicated. ^†^Independent *t*-test for continuous variables. ^‡^Statistically significant values (*P < *0.05) are shown in bold. ^§^χ² test for categorical variables. ^∥^Mann-Whitney test for nonparametric statistical test.

**Figure 5:**
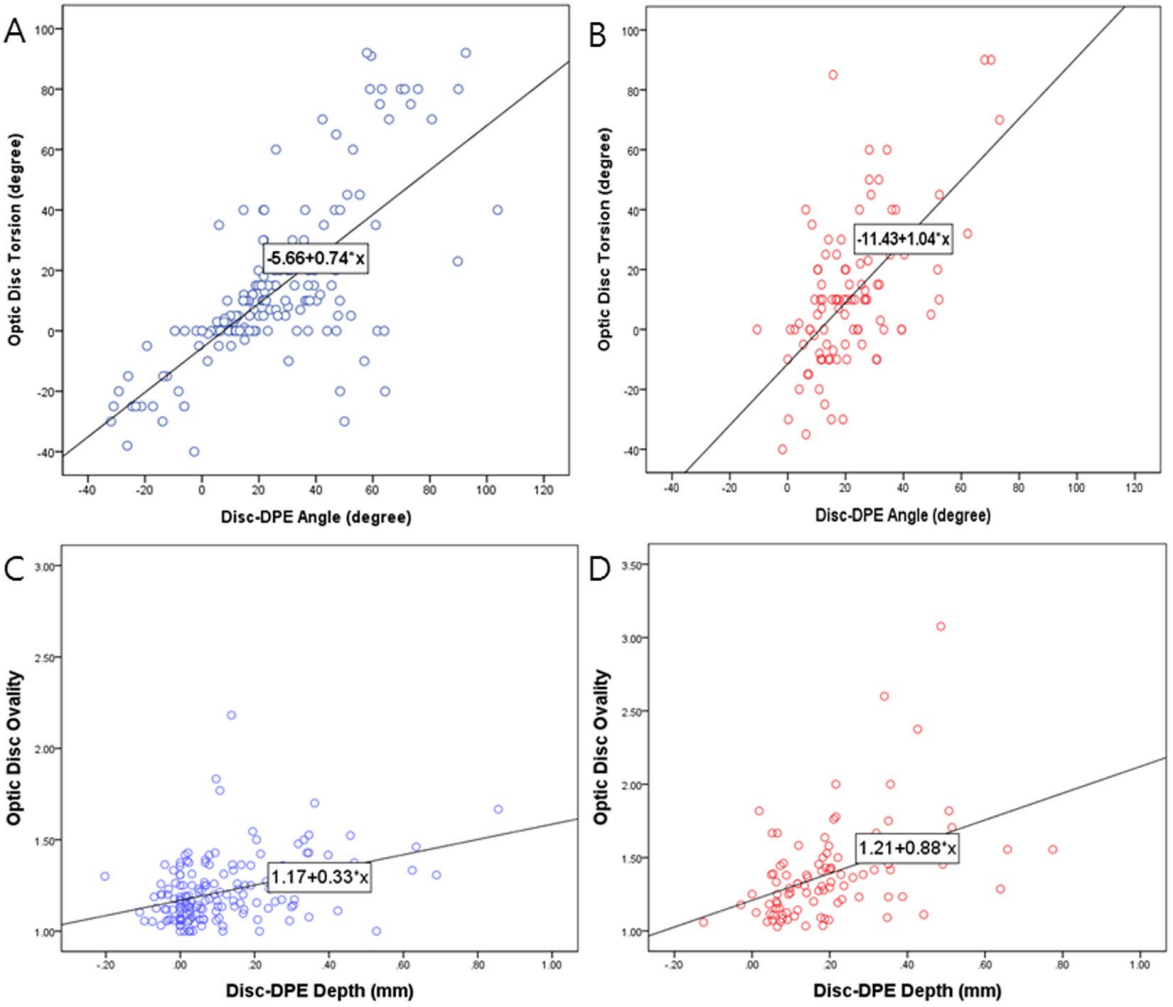
(**A**,**B**) Linear regressions result on the association of the Disc-DPE angle and optic disc torsion degree, showing a different regression coefficient between the myopes without NTG and myopes with NTG. (**C**,**D**) Linear regressions result on the association of the Disc-DPE depth and optic disc ovality, showing a different regression coefficient between the myopes without NTG and myopes with NTG.

To determine whether there was an association beyween location of the DPE and optic disc tilt and torsion, univariate and multivariate linear regressions were done separately in the myopes without NTG and myopes with NTG (Table 6). In univariate analysis in myopes without NTG, the degree of optic disc torsion increased (became more positive) with increasing fovea-disc depth (β = 31.44, *P* = 0.001) and Disc-MPP angle (β = 0.740, *P* < 0.001). In multivariate regression analysis in myopes without NTG, larger disc-DPE angle (β = 0.740, *P* < 0.001) was the strongest predictor of optic disc torsion. While univariate and multivariate analyses of optic disc torsion in myopes with NTG had similar results, this group had a larger regression coefficient (β = 1.035, *P* < 0.001) than the myopes without NTG (Fig. 5). In univariate and multivariate regression analyses in myopes without NTG, increasing disc-DPE depth (β = 0.334, *P* < 0.001) was the strongest predictor of disc ovality. Univariate and multivariate analyses on the myopes with NTG also revealed that increasing Disc-DPE depth had the strongest association with disc ovality, but had a larger regression coefficient compared to myopes without NTG (β = 0.883, *P* < 0.001) (Fig. 5).

**Table 6:** Univariate and multivariate regression analyses of optic disc torsion and disc ovality on myopic non-glaucomatous eyes and myopic glaucomatous eyes*.

	Optic Disc Torsion (Myopes without NTG n = 125)				Optic Disc Torsion (Myopes with NTG, n = 129)			
	Univariate Analyses		Multivariate Analyses*		Univariate Analyses		Multivariate Analyses*	
	Beta	*P* Value	Beta (95% CI)	*P* Value	Beta	*P* Value	Beta (95% CI)	*P* Value
Disc-DPE distance, per 1 mm larger	0.206	0.916			−1.838	0.348		
Disc-DPE depth, per 1 mm larger	1.445	0.923			4.938	0.748		
Disc–DPE angle,, per 1° larger	0.740	**<0.001** ^**†**^	0.740 (0.597 to 0.882)	**<0.001** ^**†**^	1.035	**<0.001** ^**†**^	1.035 (0.767 to 1.302)	**<0.001** ^**†**^
	**Disc Ovality index (Myopes without NTG n** = **125)**				**Disc Ovality index (Myopes with NTG, n** = **129)**			
	**Univariate Analyses**		Multivariate Analyses*		**Univariate Analyses**		Multivariate Analyses*	
	**Beta**	***P*** **Value**	**Beta (95% CI)**	***P*** **Value**	**Beta**	***P*** **Value**	**Beta (95% CI)**	***P*** **Value**
Disc-DPE distance, per 1 mm larger	0.035	**0.005** ^**†**^			0.081	**<0.001** ^**†**^		
Disc-DPE depth, per 1 mm larger	0.334	**<0.001** ^**†**^	0.334 (0.150 to 0.519)	**<0.001** ^**†**^	0.883	**<0.001** ^**†**^	0.883 (0.594 to 1.173)	**<0.001** ^**†**^
Disc–DPE angle,, per 1° larger	0.000	0.737			0.000	0.694		

CI = confidence interval, MPP = most protruded point. *Variables with *P* < 0.05 in univariate analyses were included in multivariate analyses. ^†^Statistically significant values (*P < *0.05) are shown in bold. The English in this document has been checked by at least two professional editors, both native speakers of English.

### Representative case

**Figure 6:**
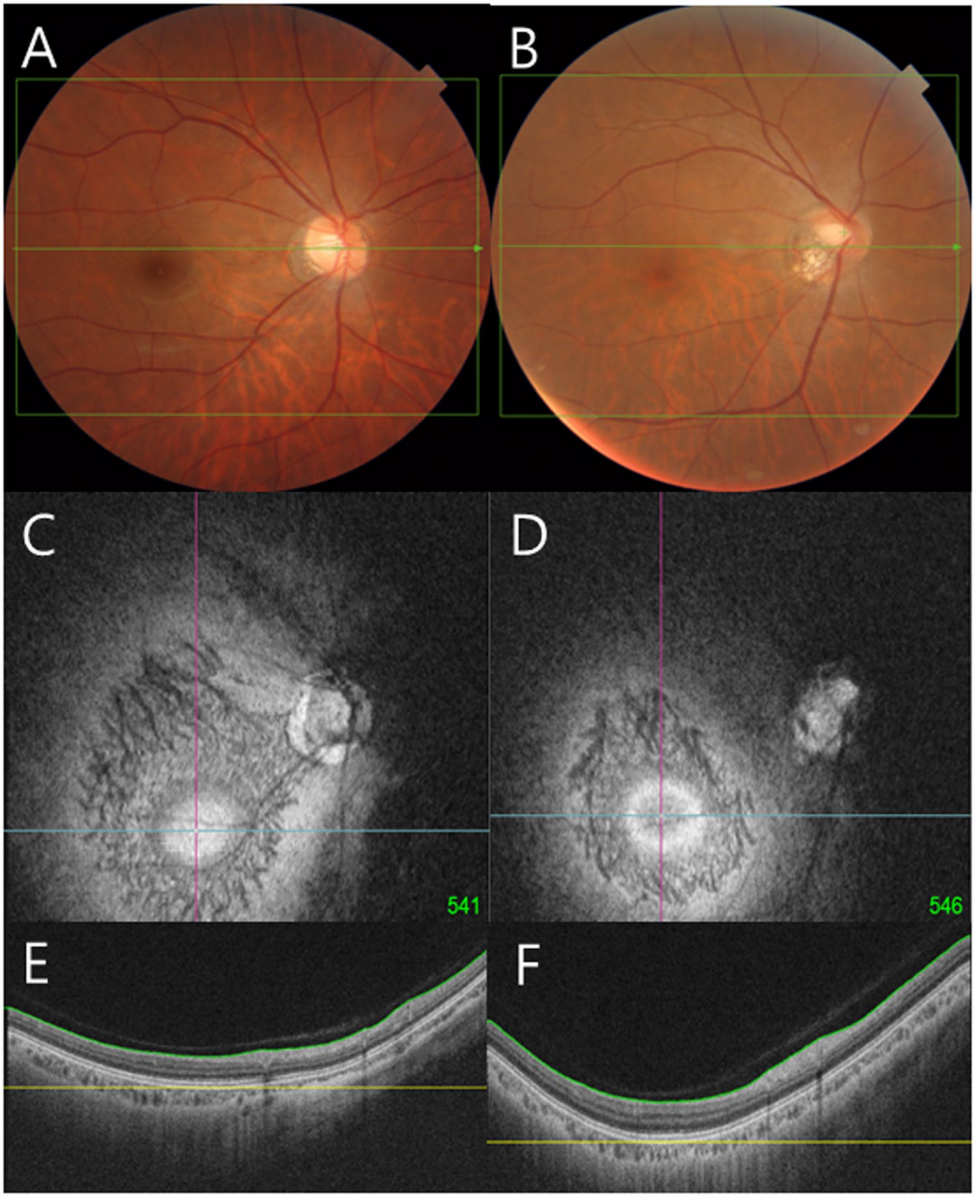
Optic disc photographs and corresponding coronal image of the DPE of subjects with similar age and axial length. (**A**,**C**,**E)** are the images of myope without NTG and (**B**,**D**,**F**) are the images of myopes with NTG. The Disc-DPE angle and the optic disc torsion are similar (A, B), but the Disc-DPE distance (4.3 mm vs 4.8 mm), and the Disc-DPE depth (0.16 mm vs 0.35 mm) shows significant difference in posterior pole configuration (**E**,**F**).

Figure 6 shows the optic disc photographs and corresponding coronal image of the DPE of subjects with similar age and axial length. Figure 6A,C,E are the images of myope without NTG and Fig. 6B,D,F are the images of myopes with NTG. The Disc-DPE angle and the optic disc torsion are similar (Fig. 6A,B), but the Disc-DPE distance (4.3 mm vs 4.8 mm), and the Disc-DPE depth (0.16 mm vs 0.35 mm) shows significant difference in posterior pole configuration (Fig. 6E,F). The larger disc tilt and larger peripapillary atrophy (PPA) shown in the myope with NTG might be the resultant of the difference in the posterior pole configuration.

## Discussion

We describe a method that determines the posterior pole from a 3D perspective. DPE was assessed as a surrogate of posterior pole configuration, which provides an additional anterior-posterior perspective. The DPE position correlated strongly with optic disc tilt and torsion than the parameter axial length in myopes with and without NTG. The deeper and more distant position of the DPE may have implications for the larger optic disc tilt and torsion characteristic of myopes with NTG. To the best of our knowledge, this is the first attempt to compare myopes with or without NTG using the parameters concentrating the posterior segment rather than using the parameters involving the whole globe.

One of the key features of myopia is an excessive axial elongation mainly in the posterior part of the eyeball. Like other organs in the human system, abnormalities may be manifested in a variety of ways. Variation in the shapes of myopic eyes presents a challenge to elucidate the pathophysiology of myopic progression. Therefore, this study concentrated on the changes in the posterior pole and its subsequent optic disc configuration alteration to identify features of the myopic eye.

The Beijing Eye Study examined the degree of myopia that is associated with a higher prevalence of glaucomatous optic nerve damage^2,29^. They reported that myopes with −6 to −8 diopters had higher prevalence than the myopes with less than −8 diopters. Such a finding is confusing because one would assume that higher degree of myopia alters the posterior pole and subsequently the ONH with a higher rate. Our data clearly demonstrates that the same degree of myopia with respect to spherical equivalent of axial length have different posterior pole configuration. The two groups were recruited by matching axial length, and the Disc-DPE distance and the Disc-DPE depth were both statistically larger in myopes with NTG. This speculation is consistent with the data showing no significant correlation between axial length of the eyeball and with the posterior pole configuration. Moreover, the axial length was the least associated with ONH configuration of myopes with NTG. Thus, our data suggests that the parameter DPE may provide a proper standard to analyze the posterior pole of myopes than the parameter axial length.

The parameter DPE further validates the proposed paradigm of the posterior pole shape alteration by Kim *et al*.^30^. The temporal crescent or the PPA is generally considered to be acquired and the consequence of scleral stretching towards the temporal direction^31,32^. Our study uses the Disc-DPE distance as a surrogate for scleral stretching in the xy plane of the Cartesian coordinates and the Disc-DPE depth as a surrogate for scleral stretching in the z axis of the Cartesian coordinates. Our data clearly demonstrates that the both parameters are associated with ONH configuration and provides an additional framework of comprehending the scleral stretching from a 3D perspective.

Our observations were extended by comparative analyses between myopes with NTG and myopes without NTG. Prominent optic disc torsion has been documented in myopic glaucomatous eyes^33,34^. While both groups in our study showed a strong association of the Disc-DPE angle and the optic disc torsion, the gradient of the coefficient was higher but the extent of the correlation was less in the myopes with NTG compared to the myopes without NTG. These data may suggest that there could be different properties of the posterior sclera that might result in the greater optic disc torsion observed in the glaucomatous eyes compared to non-glaucomatous eyes. Biomechanical findings which showed the human glaucoma eyes as well as experimental mouse and monkey glaucoma eyes were stiffer than age-matched controls also may have implications with our finding^35,36^.

**Figure 7:**
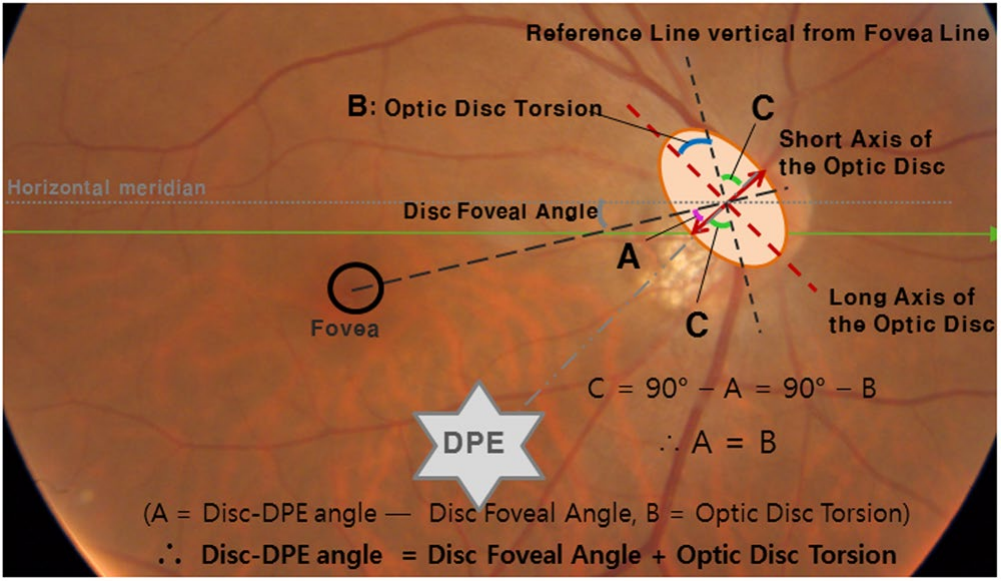
Geometric calculation on the degree of the angle of the the Disc-DPE angle as the sum of the degree of optic disc torsion and the disc foveal angle.

Asymmetric retro-equatorial elongation of the sclera would alter the location of the deepest point of the eyeball. For instance, the elongation in the superior region of the retro-equatorial sclera makes the deepest point of the eyeball inferior to the fovea. This configuration change in the posterior sclera will likely induce optic disc torsion in the positive (inferotemporal or counter-clockwise) direction. Our data suggest that degree of optic disc torsion had a strong association with DPE location (Disc-DPE angle) in the coronal section in myopic eyes, for eyes both with and without glaucoma. This assumption was confirmed by a simple geometric calculation (Fig. 7). The optic disc is leaned towards the deepest point of the eyeball, resulting in an oblique plane, on which the temporal border is located posterior to the nasal border. If the structure is imaged in an oblique plane, a two-dimensional assessment of the image will lead to an underestimation of the real size^37^. Thus, if one assumes the DPE location inferior to the optic disc, the optic disc diameter will be shortest at the same angle where the extension line of the DPE is located. In measuring optic disc torsion, we measure the deviation of the long axis of the optic disc from the perpendicular line from a horizontal line connection from the fovea. The long axis of the optic disc and the short axis of the optic disc are perpendicular to each other; likewise, the extension line from the fovea to the disc and the reference line for the disc torsion measurement are also perpendicular to each other. If four different line constituted in two right angle shares a same central angle point, geometric calculation of an angle made of any of two lines can be made. Thus, on the supposition that the two right angles share the same central point as the center of the optic disc, an equation that the Disc-DPE angle is the sum of the optic disc torsion and the degree of the disc foveal angle can be made (Fig. 7). Subgroup analyses according to the different locations of the DPE showed similar results with this geometric equation. The myopes without NTG with the DPE located near the fovea showed a disc to foveal angle of 6.45 ± 3.09°, a disc torsion angle of 7.18 ± 13.58°, and a Disc-DPE angle of 12.97 ± 9.42°. The myopes with NTG with the DPE located in the inferior hemisphere showed a disc to foveal angle of 9.17 ± 3.45°, a disc torsion angle of 27.09 ± 29.18°, and a Disc-DPE angle of 37.78 ± 15.86°.

The current study has several limitations. We employed strict inclusion criteria to assess the posterior pole profile and optic disc configuration including the exclusion of high myopia longer than 30.0 mm axial length. This might have excluded pathologic myopic eyes which should be a great concern for the clinicians. Further study is needed to examine the aspects of these severely high myopias. Second, we are unable to demonstrate conclusively that the axial elongation alters the deepest point of the eyeball because of the retrospective study design. Only the association discovered with assumption of the causality can be reported. Third, the DPE position is stable only when the subject is fixating at the scanning light. Nonetheless, there was excellent reproducibility on 30 independent series of intervisit reproducibility tests conducted on different days. Every ophthalmologic modality that scans the retina assumes that the subject is fixating the scanning light. Although the DPE is a new method that needs to be verified, just like any other structures of the fundus photograph, DPE position is reproducible as long as the proper fixation is achieved. Lastly, using number of coronal sections as a depth parameter can only be used as a surrogate. This measurement cannot be used as a real scale because of the attenuation of the signal in the nontransparent tissues^38^. However, this surrogate can function as a scale to compare between different individuals.

In conclusion, we documented a method to provide a 3D perspective of the posterior pole. The position of parameter DPE showed better association with the ONH configuration than the axial length. The deeper and more distant position of the DPE may have implications for the larger optic disc tilt and torsion characteristic of myopes with NTG. These data suggest that the DPE position may play a role in the analysis of myopes.
